# Winter movement patterns of a globally endangered avian scavenger in south-western Europe

**DOI:** 10.1038/s41598-020-74333-0

**Published:** 2020-10-19

**Authors:** Jon Morant, José María Abad-Gómez, Toribio Álvarez, Ángel Sánchez, Iñigo Zuberogoitia, Pascual López-López

**Affiliations:** 1 Department of Ornithology, Aranzadi Sciences Society, Zorroagagaina 11, 20014 Donostia-San Sebastián, Spain; 2 Conservation Biology Research Group, Department of Anatomy, Cell Biology and Zoology, Faculty of Sciences, University of Extremadura, 06006 Badajoz, Spain; 3 Servicio de Conservación de la Naturaleza y Áreas Protegidas, Junta de Extremadura, Av/ luis Ramallo s/n, 06800 Mérida, Badajoz Spain; 4 Estudios Medioambientales Icarus S.L, C/San Vicente 8, 6 ª Planta, Dpto 8, Edificio Albia I, 48001 Bilbao, Bizkaia Spain; 5 Movement Ecology Lab, Cavanilles Institute of Biodiversity and Evolutionary Biology, University of Valencia, C/ Catedrático José Beltrán 2, 46980 Paterna, Valencia Spain

**Keywords:** Animal migration, Behavioural ecology, Biodiversity, Conservation biology

## Abstract

Partial migration, whereby some individuals migrate and some do not, is relatively common and widespread among animals. Switching between migration tactics (from migratory to resident or vice versa) occurs at individual and population levels. Here, we describe for the first time the movement ecology of the largest wintering population of Egyptian Vultures (*Neophron percnopterus*) in south-west Europe. We combined field surveys and GPS tracking data from December to February during four wintering seasons (2014–2018). The wintering population consisted on average of 85 individuals (range 58–121; 76% adults and 24% subadults). Individuals were counted at five different roosting sites located near farms, unauthorized carcass deposition sites and authorized carcass deposition sites. Our results show that vultures tend to remain close to the roosting site. Moreover, we observed that females exhibited smaller home range sizes than males, which suggests a possible differential use of food sources. Overall, birds relied more on farms than other available food resources, particularly subadult individuals which exploited more intensively these sites. Our results showed that Egyptian Vultures congregate in significant numbers at specific sites throughout the winter period in south-west Spain and that these roosting and feeding sites should be given some level of legal protection and regular monitoring. Furthermore, predictable food sources might be driving the apparent increase in the non-migratory population of Egyptian Vultures, as observed in other avian species which are also changing their migratory behavior.

## Introduction

Movement is essential for most organisms in at least one stage of their life cycle, and extends across multiple spatiotemporal scales^1^. Animal movements are highly variable, from daily short-distance foraging movements to long-distance movements during some stages of their life, such as juvenile dispersal. Among them, migration is an integral part of the annual cycle of many species and is one of the most studied movement patterns from invertebrates to mammals^2^. Migration is typically thought of as a life history strategy shared by entire populations or species. Partial migration, the most common form of migration, is found across a wide variety of taxa and is more widespread in birds^3^. That is, some individuals overwinter within their breeding region (resident individuals) while others display migratory behaviour (migrant individuals) to reach distant wintering quarters^4^. Frequently, the coexistence of these migration behaviours appears to be driven by individual asymmetries in variables such as sex, age, body size, as well as by environmental conditions^5^. Furthermore, switching between these two strategies could occur at population and individual level, depending on season^6^, migration direction^7^, route, timing^8^, and distance to wintering grounds^9^.

Understanding the causes and consequences of changes in migratory behaviour is necessary to better predict population structure and dynamics (e.g., influence on survival, extent of migratory connectivity, or response to changes in breeding and non-breeding environments)^10^. The causes that lead to these changes in migratory behaviour are well known including environmental changes via phenotypic flexibility^11^; shifts in phenology through changes in inherited genetic or epigenetic pathways^12^; habitat redistribution^13^; developmental plasticity^14^ and changes in abundance/availability of food resources^15^. Among them, anthropogenic changes are known to affect the movement ecology and behaviour of long-lived species through the provision of abundant and spatially stable food subsidies^16^. For instance, this can cause several behavioural changes at individual and population levels, including dietary shifts, changes in foraging techniques and changes in social systems to find food, and also affects individual fitness and survival^15^. However, there is a lack of knowledge about the consequences of changing migratory patterns (from migratory to resident) in species with overlapping breeding and non-breeding grounds. In particular, little is known about how species behave in those environments during winter through the study of movement patterns across time and space and on the impacts of the utilization and availability of predictable food resources on daily movement patterns and migratory behaviour. Whether animals are resident or migratory has major consequences for interactions and processes in local environments^17^. In fact, human-induced changes, and the effects of climate and land-use changes in animal movement patterns have been linked to population declines in migratory species worldwide^18^.

Nowadays, thanks to the emerging use and rapid improvements in telemetry techniques^19^, we are able to determinate variations in migratory strategies^20^, and disentangle interactions between animals and their abiotic and biotic environment^21^. The integration of these techniques with traditional approaches (e.g., population monitoring) could help to better understand which factors underlie ecological and evolutionary processes in migration ecology and integrate them in conservation and management decisions. Basic movement parameters, BMPs hereafter, are used to describe movement paths (see^22^) as well as to identify common movement patterns^23^. Likewise, the use of analytical methods to assess space use (i.e., resource utilization functions; hereafter RUFs) are of great utility to identify which factors underlie those patterns from a mechanistic perspective^24^. One of the most used parameters to asses space use and resource selection is the home range estimator. Home range is the direct result of movement driven by habitat selection and other external factors, biotic interactions, and intrinsic factors related to individual state^25^. In addition, RUFs, which basically consist on a multiple regression analysis that accounts for spatial and temporal autocorrelation of tracking data^24^, are a reliable method to (1) define the fidelity to a site according to space use and sites of ecological significance in the life history of animals^26^; (2) check availability and distribution of resources^27^; and (3) to improve inference on the spatial factors influencing behaviour^28^.

The use of indicators (i.e., BMPs) gains significant relevance on the study of space use in populations of long-lived vertebrates with an altered migratory pattern^29^. Therefore, understanding how populations with altered migratory patterns spatially behave in anthropogenic environments is crucial for their conservation and management^30,31^. Vulture species have shown high behavioural plasticity with regards to local habitat structure and resource availability^32^. Furthermore, they are adequate ecological indicators and the differences in movement patterns within and between populations could help to understand complex ecological associations^30,31^. In this context, the BMPs and space use estimators are an essential tool to (1) disentangle movement patterns over time and underlying factors, (2) unravel the determinants of space use, and (3) detect highly used trophic resources by vultures’. This results in benefits to wildlife managers aimed at reducing vulture-related conflicts^33^ and conservation of these species.

The spatial ecology of the Egyptian Vulture (*Neophron percnopterus*) is still poorly known^34^, and most of the studies have focused on pure migrant or pure resident (i.e., insular) populations^26,31^. In this study, we describe for the first time the spatial ecology and resource use of a unique Egyptian Vulture population which, contrary to the commonest migratory pattern, winters in south-west Europe (instead of migrating to the Sahel region of Africa), by means of the combination of field surveys and telemetry information. To this end, we firstly describe the overwintering population size and its variation over time. Secondly, we test if vultures’ movement, extracted from BMPs, depend on both individual characteristics (i.e., age and sex) and temporal variation on environmental characteristics within the wintering season. Likewise, we test the use of different predictable food resources at fine-scale thoroughout the wintering season. Finally, we characterize the determinants of space use and identify primary drivers of vultures resource utilization through RUFs.

## Materials and methods

### Study species

The Egyptian Vulture is a medium-sized, territorial scavenger distributed from Western Europe to India and South Africa, and is globally listed as Endangered by the IUCN^35^. As much as 40% of the European breeding population is found in Spain^36^. The European breeding population is estimated at around 3000–4700 pairs^37^. It is a migratory bird that abandons its European breeding areas between late August and February^38^. The species exhibits high migratory connectivity at large spatial scales, but very diffuse migratory connectivity within subpopulations, with wintering ranges up to 4000 km apart for birds breeding in the same region, and each subpopulation visiting up to 28 countries^38^. European populations winter in sub-Saharan Africa and the Arabian Peninsula, with juveniles often remaining in the winter range for more than a year after their first migration^39,40^. Apart from these mainland populations, there are also sedentary populations inhabiting in Mediterranean islands (i.e., Menorca) and Macaronesian islands (Canary Islands, Cape Verde), and non-migratory breeding populations in sub-Saharan Africa^37^. Moreover, records from wintering individuals exist in southern Spain since mid-eighties^41^, and more recently, one young and two adults were observed in Sicily (Italy) during the wintering season 2015–2016^42^. During the twentieth century, the population of this long-lived scavenger has steadily declined across large parts of its European and African range, mainly due to unnatural mortality caused by poisoning and electrocution^35,43^. However, the survival rates are known to be higher in sedentary populations^44^.

Like other vulture species, the Egyptian Vulture presence is usually bounded to landscapes where livestock farming practices are usual^26^, as well as those regions where traditional pastoralism is still present^45^. Although Egyptian Vulture also feeds on wild prey^46^, livestock is also frequently highlighted as a cornerstone in Egyptian Vulture conservation, with the decline in extensively bred livestock considered a critical threat^35,45^. The species is listed as Vulnerable at both national and regional levels according to Spanish environmental legislation. The Egyptian Vulture population remained stable in the study area between 2008 and 2016, including 143–155 breeding pairs (data provided by the regional government, Junta de Extremadura), which represents approximately 13.6% of the Spanish population^36^.

### Study area

**Figure 1:**
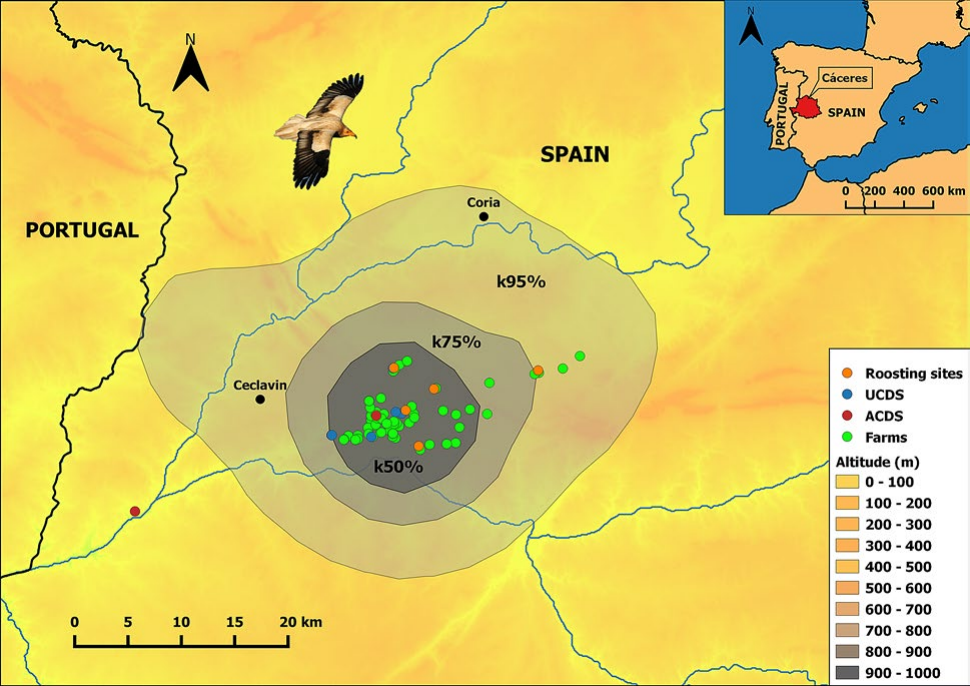
Location of the study area (upper right) including wintering roosting sites during 2014–2018, unauthorized carcass deposition sites (UCDS), authorized carcass deposition sites (ACDS), and farms. The shaded areas show three different kernel density isopleths levels derived from all individuals corresponding to 50%, 75%, and 95%, respectively. The black dots represent major towns for spatial context. The present map was done by using QGIS 3.8.3 desktop version (https://qgis.osgeo.org).

The study area is located in the western Iberian Peninsula and covers 1750 km^2^, corresponding to the administrative region of Cáceres (Extremadura, Spain) (Fig. 1). The climate is typically Mediterranean semi-arid to dry sub-humid with some oceanic influence with mild winter temperatures and autumn rainfall^47^. Average monthly temperatures are mild, but absolute minimum temperatures easily reach negative values in winter months when frosts are frequent (range 4–7 °C)^47^. Very low human population density, a markedly rural environment, and scarce industrial activity define the region, which is also recognized as one of the major biodiversity hotspots of the Mediterranean region^48^. The Natural Protected Areas network and the Natura2000 network cover 6.9% and 31% of the region, respectively^49^.

Landscapes are mostly characterized by the so-called “dehesas” (sometimes referred to as the “Spanish savannah”), agrosilvopastoral systems composed by holm oak (*Quercus ilex*) and corn oak (*Quercus suber*) forests which were progressively thinned until forming wood-pasture used for animal grazing and foraging plus crop production. Most of the region’s land is used for agriculture, combining arable and extensive livestock rearing. Overall, the livestock numbers maintained over time with slight variations. In 2005, a total of 504,908 cows, 1.6 million sheep, 174,608 goats, and 206,897 pigs whilst in 2018, a total of 592,546 cows, 1.2 million sheep, 138,291 goats, and 154,585 pigs were censused in the study area (data provided by the regional government, Junta de Extremadura). The livestock carcass disposal in the study area is allowed according with the EU legislation and regulation policies (CE 142/2011; Royal Decree 1632/2011). Moreover, CE 830/2005 made the requirements to dispose carcasses for feeding vultures at authorized feeding points more flexible, and the prohibition on carcass disposal was unofficially lifted^50^.

### Vulture capture and tagging

From September 2015 to January 2017, we trapped 12 Egyptian Vultures (2 adult males, 1 subadult male, 4 adult females, and 5 subadult females) with remotely triggered cannon nets in the surroundings of their main roosting sites in NW Cáceres. All captured individuals were ringed with yellow alphanumeric plastic and metal rings and fitted with 48 g solar-powered GPS/GSM transmitters (E-obs GmbH, Munich, Germany). Tracking devices include a GPS providing geographical coordinates, altitude, speed, bearing, and tridimensional accelerometry. Tags were programmed to record fixes (i.e., GPS positions) at 5 min intervals from 1 h before dusk to 1 h after sunset. Also, when battery levels were above a threshold of 3950 mV, GPS devices recorded locations at 1 Hz resolution (i.e., 1 location/s) during 15 min intervals called “super-bursts.” All device units were attached as backpacks using a 0.55″ (14 mm) wide Teflon ribbon harness. The weight of the transmitters and rings was 64 g, thus being below 3% of the bodyweight (mean body mass = 2176 g; range 1950–2650 g; n = 12), i.e. below the recommended limits to avoid adverse effects (i.e., 3% body mass threshold, see, e.g.,^51^).

Vultures were tracked throughout the annual cycle. For this study, we subset data to retain only information corresponding to the overwintering period (1st December to 28th February), according to the average dates of Egyptian Vulture migration in Spain^52,53^ and our field experience. According to Onrubia^54^, pre-breeding median passage time at the Strait of Gibraltar is 8th March with 95% confidence interval ranging from 20th February to 9th May; and post-breeding median passage time is 8th September with 95% confidence interval ranging from 23rd August to 24th September. Given that many migratory adults have already started their northward migration in January/February (i.e. there could be a movement towards breeding sites any time from January), we first visually inspected movements to breeding areas (if any) to ensure that none of the tagged birds exhibited territorial/breeding behaviour. After this previous exploratory analysis, we considered 1st December to 28th February as a conservative approach to include only actual wintering birds in our study. In order to homogenize the resulting dataset, we resampled locations at 5 min intervals and removed high-frequency locations (i.e., super-bursts period). Data were downloaded and incorporated automatically to the online Movebank data repository (www.movebank.org) and are publicly available upon request.

### Wintering roost sites identification and population monitoring

The wintering population was monitored monthly from December to January (two censuses per wintering period each year) between 2014 and 2018. Censuses were conducted at dusk using continuous focal sampling methods at a secure distance to avoid any disturbance to the birds^55^. Adults and subadults were classified according to plumage characteristics. We also identified food resource types where vultures were observed feeding during the sampling period in the surroundings of the wintering roosting sites. We classified food resources into three different categories: farms, authorized carcass deposition sites (hereafter ACDS), and unauthorized carcass deposition sites (hereafter UCDS). UCDS were those points close to farms where farmers released carcasses to the field without sanitary control.

### Basic movements parameters estimation and use of predictable food resources

We calculated a set of basic movement parameters (BMPs) of the tagged individuals over three wintering seasons, namely: home range size (km^2^), cumulative distance (km), intensity of use, straightness and net squared displacement (km^2^) (further details in Supplementary material Table S1). We obtained all metrics for each 15-day interval period (fortnight) of each wintering season, calculating the mean of each parameter for each individual/fortnight combination (n = 115 individual/fortnight combinations). Home range size were obtained from the 95% kernel density estimation (KDE) by using “rhrKDE” function of the “rhr” package for R^56^. The other movement parameters (see above) were derived by the “amt” package^22^.

We also calculated the proportion of non-roost GPS locations for each individual and fortnight that fell within a 300 m buffer distance to farms, UCDS, and ACDS. We selected this measurement because farmers could drop carcasses at variable distances. Buffers were generated by using “geoprocessing tool” function implemented in QGIS 3.8.3^57^.

### Modeling space use

In order to asses vultures’ space use, we used a modeling approach based on RUFs^24,58^. RUFs are often used to understand how species are related to landscape characteristics by measuring the intensity use of resources available in space, which shape the environmental niche of species (e.g., food availability, land-use, human disturbance, and topography, among others)^58^. Furthermore, one of the main advantages of the RUF method is that it accounts for spatial autocorrelation by incorporating a Matern correlation function^24^. According to Marzluff et al.’s^24^ approach, we calculated the Utilization Distribution (UD) defined as the spatial probability distribution that gives rise to a spatial point process (i.e., the recorded telemetry locations^58^). We obtained UD values from 95% KDE. To assess resource selection, we selected a set of environmental variables illustrative of the foraging habitat and ecological requirements of the species (see electronic supplementary material Table S2). We set a spatial resolution of 200 m for environmental predictors (i.e., topography, land-use and productivity). In the case of livestock density and human disturbance, we rasterized and downscaled to 200 m spatial resolution the data from polygons at each municipality level. Additionally, given that defining the overall spatial extent for resource selection studies is often subjective^59^, we established our maximum extent unit as the maximum home-range-scale determined by kernel contour volume of 95% (kernel 95%), which in turn represents landscape characteristics^24^.

### Space use estimators

Data were partitioned by individual and wintering season. We computed 95% kernel density contours for each individual to generate the UD using “rhrKDE” function of the “reproducible home range” (rhr) package for R^56^. We estimated the reference bandwidth, which defined the extent of the UD, using the “href” function implemented in the “rhr” package. UD values ranged from 0 to 95% according to kernel density estimators, where 0 was the lowest value of habitat use and 95 represented the highest value of space use. The UD was processed and included as a raster shapefile in a Geographical Information System (GIS) and converted into points to match covariate values to each pixel of 200 m at which environmental variables were recorded (i.e., livestock density, human disturbance, land-use, topography, and primary productivity) (see Supplementary materiall Table S2). Spatial analyses were done in QGIS 3.8.3^57^ and R version 3.5.1^60^.

### Statistical analyses

We used Linear Mixed Models (LMMs) to investigate variation in (1) BMPs and (2) the use of predictable food resources over time including fortnight, age, and sex as predictors. Sex, age and fortnight were entered in the models as fixed factors. Individual identity and wintering season were entered as a random intercept effects in all models. We entered the response variable (i.e., proportion of locations) by using square root arcsine transformation in the model of the use of predictable food resources. We further included resource type (i.e., farms and UCDS, excepting ACDS, given the lack of fixes within 300 m buffer of ACDS) as a two-level fixed factor, to investigate whether birds spent a different amount of time in the surrounding of different resource types. Significance of fixed effects was tested by a full model approach^61^. Models were fitted by maximum likelihood method (“lmer” function of “lme4” package^62^ for R version 3.5.1^60^). We visually inspected the homogeneity of variance and normality of residuals. We computed marginal and conditional R^2^ following using the piecewiseSEM R package^63^ to assess the overall explanatory power of the model (i.e., for fixed and random effects separately). Significance was tested by a likelihood ratio test (Anova, “car” package^64^). Moreover, we estimated the marginal means for each significant factor by using “emmeans” package^65^.

Before running statistical models of RUFs, we checked for the correlation between environmental variables. When two variables showed a correlation coefficient higher than |0.5|, the one with lower biological significance was removed from the analysis. (see Suppementary Material Table S3). Variables were scaled and mean-centered in the full RUF models.

Resource utilization functions were fitted using “ruf.fit” function implemented in the “RUF” package^24^.We ran full models for each individual (n = 12) including all predictor variables that could determine utilization distribution^61^. The importance of each resource to variations in the UD (i.e., the measure of resource use) was indicated by the magnitude of the standardized coefficients of the RUFs^24^. To test the consistency in the resource utilizations at the population level, we averaged coefficients and standard errors for each variable using the equations (1) and (2) from Marzluff et al.^24^ (see also Donovan et al.^66^ for a similar approach). Mean values were reported with ( ±) standard deviation, unless stated otherwise. Statistical significance was set at p < 0.05.

### Ethic statements

Capture, banding and monitoring of Egyptian Vultures were conducted under permits and following the protocols approved by the “Dirección General de Medio Ambiente (Consejería de Agricultura, Desarrollo Rural, Población y Territorio”, Government of Extremadura, licenses numbers: CN0011-17-AAN,CN0020-15-AAN) and following the protocols approved by the “Servicio de Conservación de la Naturaleza y Áreas Protegidas” (Government of Extremadura), following the approved guidelines. All procedures regarding animal manipulation and tagging were strictly performed in accordance with relevant guidelines and regulations of the “Patrimonio natural y de la biodiversidad” (Article 61 of Law 42/2007, Spanish Ministry of the Ecological Transition) and “Catálogo Regional de Especies Amenazadas de Extremadura” (Article 8 of Decree 37/2001 of 6 March).

## Results

### Population monitoring and food resources

**Figure 2:**
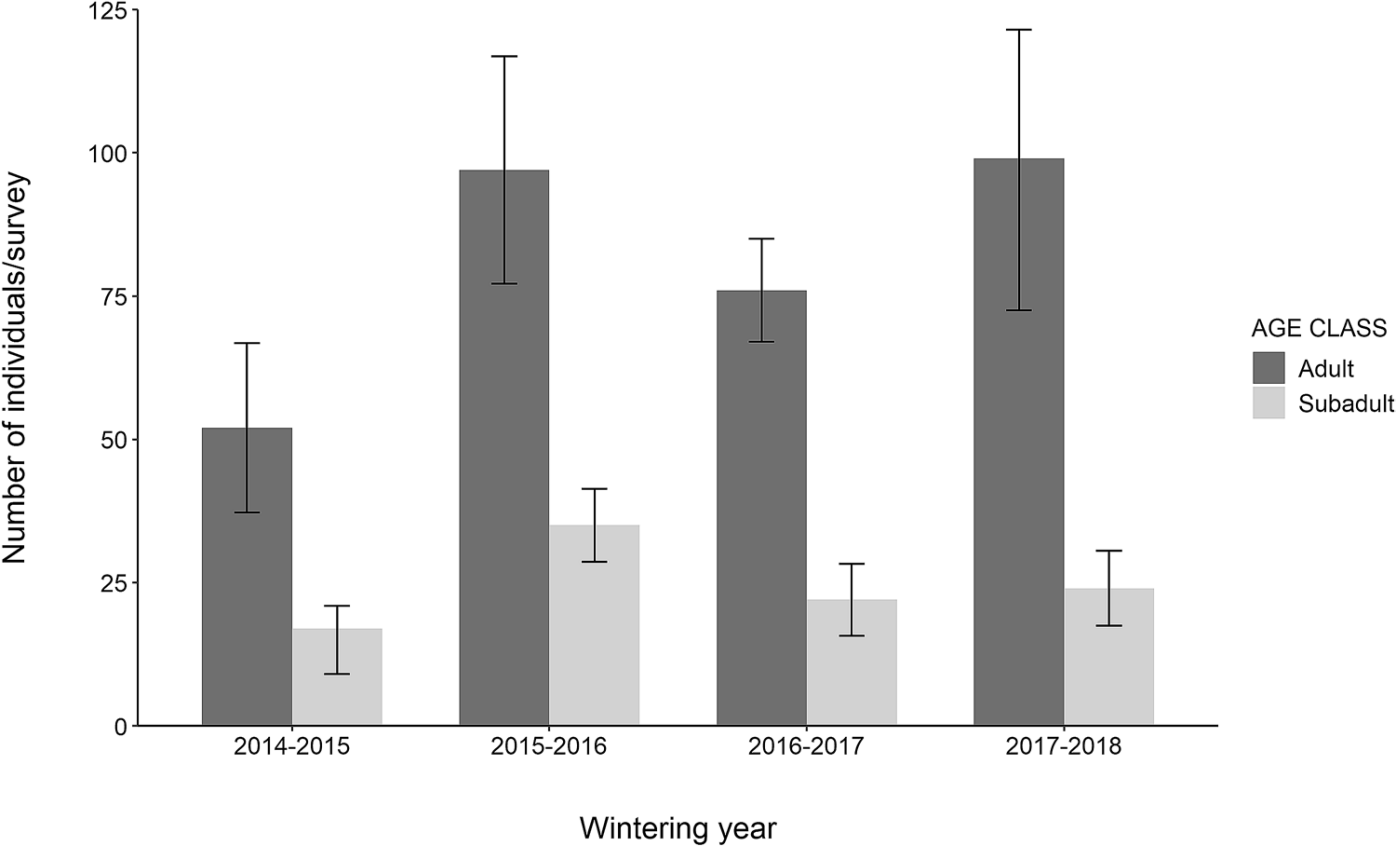
Summary of number of individuals surveyed during four consecutive wintering seasons in Cáceres (Extremadura, western Spain). The standard deviation of each age class is shown as error bars.

Five winter roosting sites were detected in the study area during four overwintering seasons (2014–2018, Fig. 1). Not all of the five winter roosting sites were simultaneously detected. In the first overwintering season (2014–2015) three winter roosting sites were identified, whilst the rest of them were detected during 2015–2016. One of the roosting sites could not be accurately surveyed because of the risk of disturbance, although the presence of wintering individuals was confirmed. Roosting sites were separated by 7.82 ± 1.34 km on average (3.32–13.52 km). On average, we counted 85 ± 10 individuals (58–121): 65 ± 10 adults (37–99) and 20 ± 2 subadults (15–25) (Fig. 2). We counted on average 6 ± 5 individuals (range 1–12) on the roosting site 1, 33 ± 26 individuals (range 6–61) on the site 2, 21 ± 15 individuals (range 5–42) on the site 3, and 27 ± 6 individuals (range 20–32) on the site 4 during the four overwintering seasons. We identified 50 farms, five UCDS, and one ACDS in the surroundings of the wintering roosting sites. The mean distance between roosting sites and the nearest farm or UCDS were 0.28 ± 0.6 km (0.14–0.48 km) and 4.24 ± 2.41 km (0.09–2.41 km), respectively. The distance between the unique ACDS within the study area and roosting sites was 6.85 ± 5.11 km (2.81–15.76 km).

### BMPs and use of predictable food resources

Our analyses were based on 123,137 GPS locations. During the study period, one of the tagged individuals lost the transmitter in the breeding area during summer 2017 (see Supplementary material Table S4). Furthermore, of all tagged individuals, two of them exhibited migratory behaviour during the following wintering season after tagging, leaving the breeding grounds to migrate to Africa. Therefore, from these individuals we only retained locations in the study for the winter season they remained in the trapping area. Overall, the mean distance between breeding and wintering the five wintering roost sites was 101 ± 121 km (20 – 345 km) for 6 of the tagged individuals. The rest of the tracked individuals (n = 6) did not breed during the study period.

The mean BMPs across individual/fortnight combinations (n = 115) were 38.66 ± 36.44 km^2^ (7.61—117.77 km^2^) for home range size, 346.03 ± 192.87 km (107.27–776.75 km) for cumulative distance, 35.51 ± 20.03 km^2^ (15.26–80.26 km^2^) for net square displacement, 0.06 ± 0.08 (0.01—0.31) for straightness index, and 25.26 ± 7.30 (11.96–33.10) for intensity of use. The mean value for the use of predictable food resources was 17 ± 0.07% (3–25%). We did not observe any location within 300 m of ACDS during the study period.

**Figure 3:**
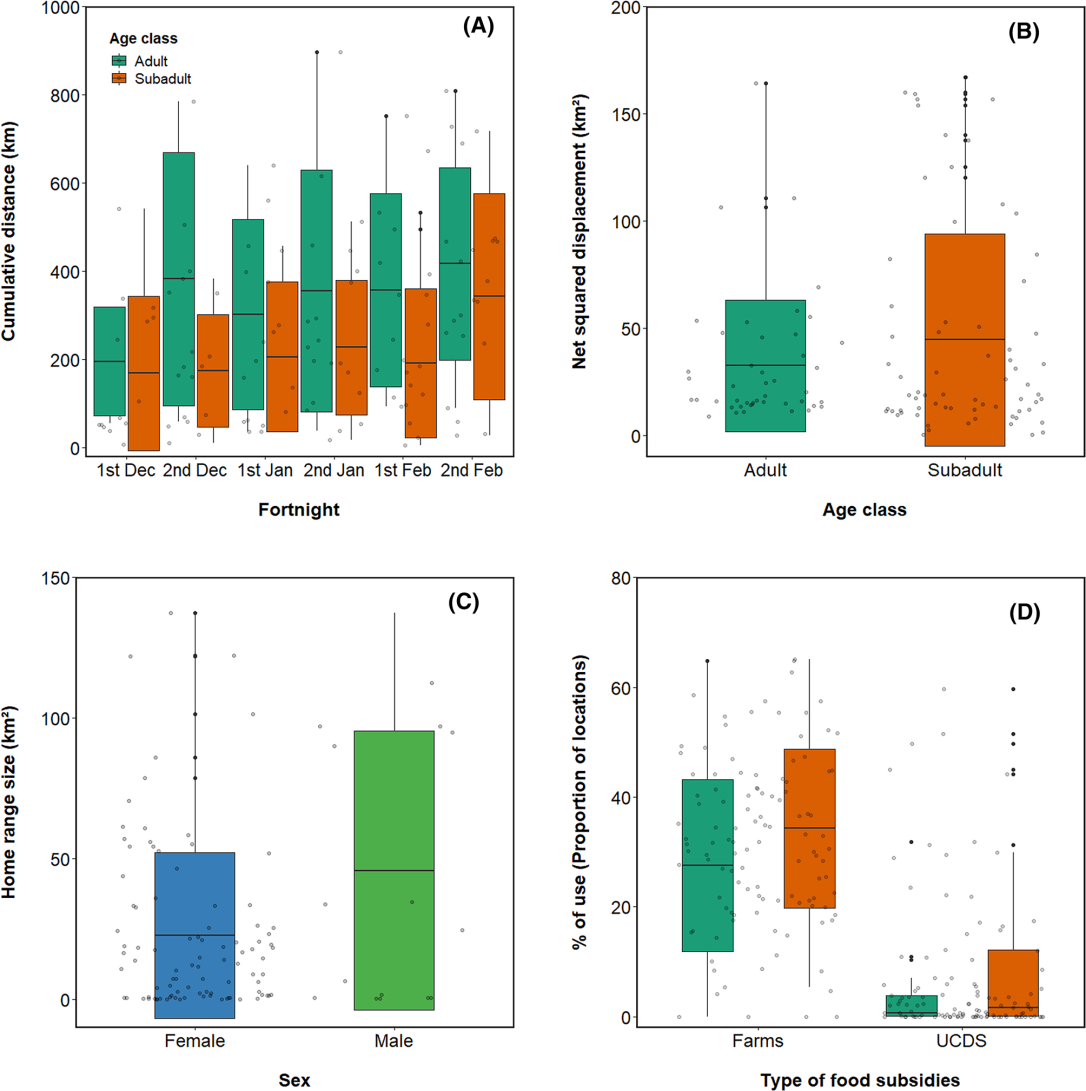
Values of the significant variables included in the full models for cumulative distance, net squared displacement, home range size and use of the different food subsidies of the tagged individuals (n = 12) corresponding to four wintering seasons (2015–2018). White dots represent the raw data points. The standard deviation is shown as error bars.

LMMs for cumulative distance and net squared displacement showed a significant effect of fortnight, with increasing movement activity through the winter (Table 1; Fig. 3). Moreover, females exhibited smaller home ranges than males (Table 1). Adult individuals showed higher values of cumulative distance than subadults. On the contrary, net squared displacement values were higher in subadults than in adults (Table 1). The variation captured by our three predictor variables (< 15%) compared with that captured by random terms was low (Table 1) for all BMPs. Only the cumulative distance model captured more than 10% of the variability in the data (18%). The results of the model for the use of predictable food resources also showed that there were differences among age groups and the type of food subsidies used by individuals (Table 1). Overall, individuals made more intensive use of farms than UCDS. Likewise, subadult individuals exhibited higher values of use of farms and UCDS than adults (Fig. 3). In this case, the variability in the data was highly captured by the predictor variables (> 50%) (Table 1).

**Table 1:** Estimates for fixed terms of full models for each BMPs and the use of predictable food resources.

Variable	Predictors	Estimate ± SE	Chisq	Pr(> Chisq)	R^2^ fixed	R^2^ random
Home range size	Age	− 10.14 ± 11.38	0.716	0.397	0.081	0.221
	Sex^a^	36.297 ± 15.834	4.984	**0.025**		
	Fortnight	1.410 ± 2.429	1.322	0.932		
	Intercept	29.176 ± 13.057				
Cumulative distance	Age^b^	–149.089 ± 50.992	8.893	**0.002**	0.183	0.546
	Sex	137.767 ± 85.389	2.637	0.104		
	Fortnight^c^	30.137 ± 7.949	19.769	**0.001**		
	Intercept	271.520 ± 71.718				
Intensity of use	Age	− 5.098 ± 3.065	2.906	0.088	0.048	0.169
	Sex	4.683 ± 4.221	1.100	0.294		
	Fortnight	0.437 ± 0.709	4.285	0.509		
	Intercept	24.533 ± 3.746				
Straightness	Age	0.047 ± 0.046	0.992	0.319	0.028	0.603
	Sex	− 0.042 ± 0.076	0.363	0.546		
	Fortnight	− 0.008 ± 0.007	4.720	0.450		
	Intercept	0.089 ± 0.048				
Net squared displacement	Age^d^	23.250 ± 9.731	5.946	**0.014**	0.090	0.180
	Sex	− 19.927 ± 13.498	2.388	0.122		
	Fortnight	5.769 ± 2.432	8.117	0.149		
	Intercept	3.749 ± 14.019				
Use of predictable food resources	Age^e^	4.403 ± 1.534	6.837	**0.008**	0.601	0.013
	Sex	− 3.965 ± 2.064	2.157	0.141		
	Fortnight	− 0.064 ± 0.384	0.329	0.997		
	Type^f^	− 0.409 ± 0.025	258.900	** < 0.001**		
	Intercept	− 1.821 ± 2.137				

Age, sex, fortnight and type of predictable food resource were coded as factors, using “adult”, “female”, “farms” and “fortnight 1” as referencence values for statistical comparison, respectively. Significant values are highlighted in bold. The variance explained by fixed (R^2^ fixed) and random effects (R^2^ random) of each full model are shown. *SE* standard error. The estimated marginal means (mean ± SE) for each significant factor are shown as table footnote*.

*^a^Home range size (km^2^) (males: 65.7 ± 16.51, females, 29.4 ± 6.69), ^b,c^cumulative distance (km) (adults: 453 ± 76.5, subadults: 304 ± 68, fortnight 1: 315 ± 75.8, fortnight 2: 328 ± 72.9, fortnight 3: 350 ± 73.7, fortnight 4: 390 ± 72.2, fortnight 5: 371 ± 71.9, fortnight 6: 481 ± 71.9), ^d^net squared displacement (km^2^) (adults: 15.4 ± 12.7, subadults: 38.6 ± 10.6), ^e,f^use of predictable food resources (adults: 8.95 ± 1.66, subadults: 13.35 ± 1.19; farms: 30.63 ± 1.48, UCDS: 4.67 ± 1.48).

### Space use

According to the population-level models for the full combination of each individual–resource category, the RUF analysis showed that the best predictors of space use at the population level were food availability, particularly goat density and cow density, to a lesser extent, as well as land use variables (forest and agriculture lands) (Table 2; Supplementary material Table S5). On the contrary, areas with high density of sheep, pigs and variables related to human disturbance such as areas close to villages and artificial surfaces were avoided.

**Table 2:** Results of the averaged coefficients $\widehat{({\beta }_{j})}$ and standard errors from the full RUFs models of the tracked individuals (n = 12) (see Supplementary Material Table S2 for details).

Variable	Estimate (*β*_*j*_) ± SE	Var (*β*_*j*_)	LCI (95%)	UCI (95%)
Sheep	− 0.407 ± 0.193	0.022	− 1.017	0.203
Pigs	− 0.318 ± 0.175	0.008	− 0.712	0.076
Cows	0.304 ± 0.295	0.036	− 0.134	0.741
Goats	2.356 ± 0.279	0.017	− 0.399	5.110
Distance to roads	0.007 ± 0.132	0.006	− 0.427	0.441
Distance to towns	− 0.310 ± 0.137	0.007	− 1.244	0.624
Forest	1.821 ± 0.504	0.128	0.513	3.129
Artificial	− 1.161 ± 0.998	0.339	− 2.786	0.463
Agriculture	1.127 ± 0.399	0.107	− 0.151	2.405
Slope	− 0.748 ± 0.161	0.018	− 1.946	0.450
NDVI	− 0.186 ± 0.089	0.003	− 0.623	0.251

*SE* standard error, *var* variance, *LCI* lower confidence interval, *UCI* upper confidence interval.

## Discussion

Our results provide the first insight into the movement patterns of the largest overwintering population of the Egyptian Vulture in south-western Europe. Population monitoring data reveals that the number of wintering individuals was 121, which were congregated in five close roosting sites throughout four wintering seasons. We observed that most birds counted in each survey/wintering season (see Fig. 2) were adults (75.98%). Yet, it remains unclear if the population is made up of subadults that do not migrate and then remain, or adults that opt to not migrate even after several successful migrations. The probability of switching migration tactic should increase with age particularly for residents^67^, but what causes the differences in wintering numbers is unknown. To date, there are only two sites in western Europe where similar behaviour had been reported, with only 20–30 individuals surveyed in the mid-eighties in the south of Spain^41^ and, more recently, three individuals in Sicily (Italy)^42^.

Communal roosting is widely distributed among animals, and some of the proposed benefits of aggregation include the exchange of information for finding food, mate acquisition, and thermoregulatory purposes^68^. In birds, especially soaring raptors that exhibit social behaviour, it is important to access safe places to rest, meet, exchange information, obtain refuge from predators, and avoid adverse weather conditions^69^. In this context, the observed distances from roosting sites to farms and UCDS (see Fig. 1) may suggest that predictable food sources may attract animals to the resting trees^31^. This could explain the small home range size found in our study (< 50 km^2^) when compared to the home range size found during the same period in a sedentary population of the species (> 100 km^2^^31^) and much smaller than that of individuals overwintering in Africa (> 9000 km^2^^40^).

We observed that home range size was affected by intrinsic factors, particularly sex. However, the poor variability captured by our models suggests that other non-evaluated factors might also be operating, such as environmental conditions or intraspecific interactions in the wintering roosting sites^70^. Furthermore, the observed results could also be due to the disparities in the sample size regarding the sex of individuals (nine females and four males). We found that females exhibited smaller home range sizes than males. This pattern could decrease intraspecific competition by food resources in the study area (farms, UCDS, and ACDS) due to the unpredictable nature of food supplies^31^.

We found differences in net squared displacement among age classes with larger values in subadults. Adults exhibited a more marked sedentary behaviour than subadults, likely due to the experience and the knowledge of the place of those predictable food sources are. The short distances between roosting sites and feeding points could be advantageous in reducing foraging distance and thus energy expenditure while increasing fuel load during winter when adverse weather conditions affect birds' flight capacity^30^. Non-experienced subadults, however, tend to move longer distances looking for predictable and also natural (unpredictable) food sources to meet energy requirements^31^. Similarly, we found that individuals travelled longer distances in the course of the winter. In this case, adult birds tended to move longer distances than subadult birds. These larger movements closer to spring could be related to the onset of the breeding season^71^, particularly for adults which, if they were migratory individuals, would be travelling 300 km per day north from their wintering ranges in Africa any time from January onwards^40^.

Individuals can vary in their use of predictable food resources according to age, sex, cultural and personality differences^15^. Here, we found that overall, subadult birds rely more on predictable food resources than adults. Likewise, both adults and subadults make more intensive use of farms than UCDS. These results suggest that the intensity of the use are driven by individual traits, particularly by social status^31^. In this context, younger bird's preference seems to be explained by their limited environmental knowledge comparing to adult individuals^72^. Although some studies showed that vultures rely more on feeding stations than on the surroundings of cattle farms^26^, our results reveal that farms play a more important role comparing to UCDS and ACDS, with the latter never being visited by the vultures tracked in this study. The observed high-intensity use of these sites may suggest that those places might have also become more predictable (and thus more stable) during winter compared to other available food resources like UCDS and ACDS (but see^73^). Likewise, it may also drive the changes, not only regarding foraging patterns, but also the establishment of resident populations as it has been seen in other species (e.g. white storks), depending on artificial food supplies^16^.

The RUF analysis showed that vultures preferred forest areas (“dehesas”) and agricultural lands with high goat density and some cows far from towns. In these places vultures benefit from feeding on newborn cattle as well as the excrements of cows, where they obtain carotenoids which are in turn essential pigments for status signaling^74^. This reflects the main landscape characteristics of the wintering area of this unique population of an endangered vulture. In fact, Extremadura’s “dehesas” represent one of the hottest hotspots of vertebrate diversity across Europe^48^. A combination of mild climatic conditions as well as biogeographical and refugia effects that occurred during the last glaciations^75^ make this area of Europe particularly favourable for the establishment of resident populations of species that were once largely migratory such as the White Stork or the Egyptian Vulture.

### Evolutionary and conservation implications

Our results showed that mainland Egyptian Vulture population should be reclassified from migratory (excluding insular populations that are resident) to a facultative partially migratory species. Gilroy et al.^10^ noted that species with more considerable within-population variability in migratory movements might be more resilient to environmental change and facilitate adaptive responses to climate change. The number of threats affecting Egyptian Vultures in their African winter quarters is increasing^76^. Therefore, a shift from a migratory behaviour to a sedentary one could have positive effects on the conservation status of the Egyptian Vulture in Europe in the long-term^77^. However, the continuous presence of some individuals in a given area implies that they are also subject to threats that are only faced during the breeding period for migratory conspecifics^38^, such as human disturbances and habitat alterations in roosting or nesting sites , collisions with wind farms and illegal poisoning^78^. Besides, the strong dependence on food resources provided by humans direct or indirectly by intensive livestock farming practices could increase poisoning risk^79^. Overall, we encourage managers and conservation practitioners to take into account the emergence of these new behaviours to ensure adequate conservation of existent or new wintering roosting sites. Furthermore, we recommend the integration of movement patterns, foraging ecology and the use of protected areas to assess species susceptibility to different threats^38^ , to better inform conservation planning, and to improve management decisions, ensuring population viability and reducing human-vulture conflicts^80^.

## Supplementary information

Supplementary Information
