# Supplementary material for: Winter movement patterns of a globally endangered avian scavenger in south-western Europe
Source: Sci Rep. 2020 Oct 19;10:17690. doi: 10.1038/s41598-020-74333-0 (PMC7572415; doi:10.1038/s41598-020-74333-0)
Supplement: Supplementary file 1 — Supplementary Information [file 41598_2020_74333_MOESM1_ESM.pdf]

Supplementary Material

Winter movement patterns of a globally endangered avian scavenger in south-western Europe

Jon Morant Etxebarria[1,*], José María Abad-Gómez[2,3], Toribio Álvarez[3], Ángel Sánchez[3], Iñigo Zuberogoitia Arroyo[1,4], Pascual López-López[5]

[1]Department of Ornithology, Aranzadi Sciences Society, Zorroagagaina 11, 20014 Donostia-San Sebastián, Spain. Phone number: +34 635988515 Email: jon_morant@hotmail.com

[2]Conservation Biology Research Group, Department of Anatomy, Cell Biology and Zoology, Faculty of Sciences, University of Extremadura, 06006, Badajoz, Spain. Email: abadjm@unex.es

[3]Servicio de Conservación de la Naturaleza y Áreas Protegidas. Junta de Extremadura. Av/ Luis Ramallo s/n 06800 Mérida (Badajoz) Spain. Email: angel.sanchezga@juntaex.es , toribio159@gmail.com

[4]Estudios Medioambientales Icarus S.L. C/ San Vicente, 8. 6 ª Planta. Dpto 8. Edificio Albia I. 48001, Bilbao, Bizkaia. Spain. Phone number: +34 636423851 Email: zuberogoitia@icarus.es

[5]Morevement ecology lab, Cavanilles Institute of Biodiversity and Evolutionary Biology, University of Valencia, C/ Catedrático José Beltrán 2, E-46980, Paterna, Valencia, Spain. Email: Pascual.Lopez@uv.es

*Corresponding author: jon_morant@hotmail.com

Table S1. Summary calculations of selected Basic Movement Parameters (BMPs) (see also [1,2] and [3] for further details).

Parameter	Unit	Calculation	Abbreviation	References
Cumulative distance	m/ km	-	Increment of the X and Y values between two consecutive relocations, change in absolute spatial position	2,4,5,6
Net squared displacement	m^2/ km^2	$NSD - VarX - VarY$	X and Y = cartesian coordinates of each point of trajectory change along the path	2,4,5,6
Straightness	Range (0-1)	$\mathrm{ST} = \frac{dE}{L}$	dE = Euclidean distance between the beginning and end of the path<br>L= Total path length	2,7,8
Intensity of use	Range (0-100)	$\mathrm{IU} = \frac{L}{\sqrt{A}}$	L = total path length<br>A = area of the movement	9,10

Table S2. Environmental variables at 200 m spatial resolution used to model Egyptian vulture space use wintering in Extremadura (western Spain) (see Methods section).

		Variable	Code	Description	Hypothesis	Resolution	Data source
Environmental Conditions	Topography	Insolation	INSA	Insolation (from 1st December to 28th February).	Determinate insolation values may favour the foraging efficiency of the species.	200m	DEM 200m http://centrodedescargas.cnig.es/CentroDescargas/index.jsp
		Slope	SDLO	Slope of the terrain (%)	Areas with lower slope and terrain roughness values favor the detection of carcasses.		
		Rugosity	TOPV	Rugosity (%)			
	Land-use	Land-cover	LANDCO	Dominant "habitat", according to the main CORINE land cover levels in each cell corresponding to Agricultural, Artificial and Forest areas.	Some habitat types may facilitate the detection of carcasses in the field.	200m	CLC 2018 GeoTIFF 100m https://land.copernicus.eu/pan-european/corine-land-cover/clc2018?tab=download
	Productivity	NDVI	NDVI	Mean anual Normalized Difference Vegetation Index (NDVI).	NDVI is a indicator of habitat quality. Higher NDVI values are associated with more productive grazing environments [11].	200m	NDVI GeoTIFF 300m https://land.copernicus.eu/global/products/ndvi
	Human disturbance	Urban nuclei density	DENSN	The inverse of the Euclidean distance the nearest urban nucleus (0 value to the greatest distance between nuclei).	The presence of low populated nuclei in rural areas may favour the presence of the species.	200m	Data obtained at municipality level http://centrodedescargas.cnig.es/CentroDescargas/buscar.do?filtro.codFamilia=REDTR#
		Distance to towns	DISTOWN	Euclidean distance to towns and villages.	The presence of towns and villages may alter foraging behaviour.		
		Distance to roads	DISTRO	Euclidean distance to asphalted communication routes (roads and highways).	The presence of roads may alter foraging behaviour [(12,13)].		
Trophic Resources	Livestock	Cows<br>Pigs<br>Sheep<br>Goats	COWS<br>PIGS<br>SHEEP<br>GOATS	Livestock density (i.e., heads of cows, sheeps, pigs, and goats divided by the surface area of each local Municipality).	Livestock is an important food resource for the species and is an adequate proxy of food availability[14].	200m	Data obtained at municipality level from the the annual regional cattle census (2017-2018) performed by the regional government (Junta de Extremadura).

Table S3. Correlation matrix of the variables used in RUF analysis based on the pooled sample of 123,137 GPS locations of the tagged individuals (n=12) (more details of each variable in Table S2). Correlated variables (>0.5) are highlighted in bold.

	INSA	SDLO	TOPV	FOREST	ARTI	AGRIC	NDVI	DENSN	DISTRO	DISTOWN	COWS	PIGS	SHEEP	GOATS
**INSA**	**1.00**	-	-	-	-	-	-	-	-	-	-	-	-	-
**SDLO**	**0.62**	**1.00**	-	-	-	-	-	-	-	-	-	-	-	-
**TOPV**	**0.74**	**0.73**	**1.00**	-	-	-	-	-	-	-	-	-	-	-
**FOREST**	0.00	0.07	0.00	**1.00**	-	-	-	-	-	-	-	-	-	-
**ARTI**	0.04	0.00	0.02	-0.08	**1.00**	-	-	-	-	-	-	-	-	-
**AGRIC**	0.00	0.04	0.00	0.01	-0.09	**1.00**	-	-	-	-	-	-	-	-
**NDVI**	0.00	0.19	0.28	-0.19	0.00	0.21	**1.00**	-	-	-	-	-	-	-
**DENSN**	0.08	0.00	0.00	-0.06	0.09	0.06	0.31	**1.00**	-	-	-	-	-	-
**DISTRO**	-0.24	-0.21	-0.27	0.07	-0.06	-0.13	-0.33	**-0.62**	**1.00**	-	-	-	-	-
**DISTOWN**	-0.08	0.00	0.00	0.06	-0.09	-0.06	-0.31	**-1.00**	0.01	**1.00**	-	-	-	-
**COWS**	-0.07	-0.2	-0.25	0.00	-0.06	0.00	-0.18	-0.09	0.27	0.09	**1.00**	-	-	-
**PIGS**	-0.1	-0.2	-0.28	0.16	0.00	-0.22	-0.34	0.13	0.04	-0.13	0.02	**1.00**	-	-
**SHEEP**	-0.1	-0.19	-0.27	0.00	0.04	0.00	-0.15	0.03	0.19	0.03	0.19	0.2	**1.00**	-
**GOATS**	-0.09	-0.21	-0.26	0.16	0.00	-0.22	-0.31	0.08	0.07	-0.08	-0.06	0.27	0.27	**1.00**

Table S4. Details of each tagged individual during study period.

Ring	Age	Sex	Tag ID	ID	Tagging year	Tracking duration (days)*	Fate of tagged individuals during study period
9071267	Adult	F	5179	Arenal	2017	396	Alive
9071263	Subadult	F	4524	Espiga	2016	760	Alive
9068408	Subadult	F	4023	Fresnedosa	2015	1111	Alive
9071040	Adult	F	4245	Lluvia	2015	802	Alive
9071039	Subadult	F	4243	Macedonia	2015	802	Alive
9071262	Adult	F	4244	Niebla	2015	802	Alive
9071268	Subadult	M	5180	Pando	2017	396	Alive
9071261	Subadult	F	4025	Primavera	2015	802	Alive
9071241	Adult	M	5181	Taiga	2017	110	Alive
9071269	Adult	M	5181	Torre	2017	146	Alive
9071242	Adult	F	5182	Villa	2017	79	Alive
9071243	Subadult	F	5183	Viña	2017	79	Alive

*****Total number of days from the first day when bird was tagged until the end of study period (28th of February 2018).

Table S5. Estimates of standardized RUF coefficients (β) and standard errors for each variable of the full RUF models for 12 Egyptian vultures tracked by GPS satellite telemetry in Extremadura (Spain). Consistency at the population level is indicated by the mean and standard deviation of each coefficient and standard error (see also Donovan et al.[15] for a similar approach). According to Marzluff et al. [16], the relative importance of resources is indicated by the magnitude (positive or negative) of β.

	Livestock				Human disturbances		Land-use			Topography	Productivity
	**Sheep**	**Pigs**	**Cows**	**Goats**	**Distance to roads**	**Distance to towns**	**Forest**	**Artificial**	**Agriculture**	**Slope**	**NDVI**
**ID**	**β ± SE**	**β ± SE**	**β ± SE**	**β ± SE**	**β ± SE**	**β ± SE**	**β ± SE**	**β ± SE**	**β ± SE**	**β ± SE**	**β ± SE**
1	-2.571 ± 0.096	-0.579 ± 0.304	2.189 ± 0.097	8.110 ± 0.356	-0.477 ± 0.080	-0.966 ± 0.077	2.777 ± 0.131	-0.128 ± 0.048	2.993 ± 0.132	-0.316 ± 0.052	0.554 ± 0.078
2	-0.025 ± 0.010	-0.372 ± 0.068	0.049 ± 0.009	0.378 ± 0.072	-0.351 ± 0.015	0.282 ± 0.014	-0.017 ± 0.004	0.016 ± 0.001	-0.023 ± 0.004	-0.055 ± 0.004	0.059 ± 0.005
3	-0.221 ± 0.032	-0.610 ± 0.254	0.165 ± 0.030	1.615 ± 0.270	-0.148 ± 0.046	-0.470 ± 0.051	-0.012 ± 0.010	0.001 ± 0.003	-0.031 ± 0.012	-0.148 ± 0.017	0.074 ± 0.015
4	0.369 ± 0.009	0.276 ± 0.011	0.787 ± 0.820	-3.567 ± 0.091	-0.945 ± 0.066	-0.967 ± 0.022	6.135 ± 0.182	-9.437 ± 5.512	6.190 ± 0.183	0.226 ± 0.007	-0.109 ± 0.012
5	0.072 ± 0.003	0.036 ± 0.002	0.056 ± 0.003	0.046 ± 0.006	-0.162 ± 0.037	-0.700 ± 0.071	-0.011 ± 0.003	-0.006 ± 0.003	0.016 ± 0.003	0.030 ± 0.003	-0.033 ± 0.003
6	0.335 ± 0.173	-0.308 ± 0.204	0.956 ± 0.279	15.127 ± 0.233	2.190 ± 0.198	2.810 ± 0.200	3.219 ± 0.401	0.780 ± 0.158	4.589 ± 0.395	-0.899 ± 0.068	-0.077 ± 0.137
7	0.039 ± 0.069	0.315 ± 0.058	-0.061 ± 0.067	-0.084 ± 0.054	-0.455 ± 0.088	-0.732 ± 0.093	-0.402 ± 0.067	0.328 ± 0.036	-0.403 ± 0.072	-0.188 ± 0.064	-0.551 ± 0.057
8	0.374 ± 0.095	-0.186 ± 0.096	-0.539 ± 0.071	0.914 ± 0.918	0.072 ± 0.091	0.912 ± 0.112	1.331 ± 0.074	0.157 ± 0.018	1.251 ± 0.081	-0.375 ± 0.050	0.403 ± 0.056
9	0.015 ± 0.010	0.074 ± 0.111	-0.013 ± 0.018	-0.077 ± 0.121	-0.089 ± 0.018	-0.072 ± 0.020	-0.035 ± 0.004	-0.023 ± 0.002	-0.034 ± 0.001	-0.018 ± 0.007	-0.232 ± 0.010
10	-2.749 ± 1.759	-2.318 ± 0.931	-0.632 ± 2.088	3.276 ± 1.167	0.415 ± 0.900	-4.355 ± 0.938	3.658 ± 4.145	-0.881 ± 1.398	-1.125 ± 3.900	-7.404 ± 1.609	-2.467 ± 0.634
11	-0.346 ± 0.027	-0.119 ± 0.026	0.792 ± 0.027	2.368 ± 0.023	0.009 ± 0.023	0.042 ± 0.024	5.248 ± 0.996	-3.956 ± 3.968	0.020 ± 0.001	0.000 ± 0.023	-0.029 ± 0.023
12	-0.176 ± 0.029	-0.018 ± 0.038	-0.105 ± 0.028	0.165 ± 0.035	0.024 ± 0.027	0.493 ± 0.027	-0.039 ± 0.029	-0.787 ± 0.824	0.081 ± 0.004	0.173 ± 0.030	0.175 ± 0.037

References

1. Almeida, P. J. A. L., Vieira, M. V., Kajin, M., German, F. M. & Cerqueira, R. Indices of movement behaviour: Conceptual background, effects of scale and location errors. *Zoologia* (2010). doi:10.1590/S1984-46702010000500002

2. Edelhoff, H., Signer, J. & Balkenhol, N. Path segmentation for beginners: An overview of current methods for detecting changes in animal movement patterns. *Movement Ecology* (2016). doi:10.1186/s40462-016-0086-5

3. Signer, J., Fieberg, J. & Avgar, T. Animal movement tools (amt): R package for managing tracking data and conducting habitat selection analyses. *Ecol. Evol.* (2019). doi:10.1002/ece3.4823

4. Calenge, C., Dray, S. & Royer-Carenzi, M. The concept of animals' trajectories from a data analysis perspective. *Ecol. Inform.* (2009). doi:10.1016/j.ecoinf.2008.10.002

5. Gutenkunst, R., Newlands, N., Lutcavage, M. & Edelstein-Keshet, L. Inferring resource distributions from Atlantic bluefin tuna movements: An analysis based on net displacement and length of track. *J. Theor. Biol.* (2007). doi:10.1016/j.jtbi.2006.10.014

6. Morales, J. M. & Ellner, S. P. Scaling up animal movements in heterogeneous landscapes: The importance of behavior. *Ecology* (2002). doi:10.1890/0012-9658(2002)083[2240:SUAMIH]2.0.CO;2

7. Gurarie, E. *et al.* What is the animal doing? Tools for exploring behavioural structure in animal movements. *J. Anim. Ecol.* (2016). doi:10.1111/1365-2656.12379

8. McKenzie, H. W., Lewis, M. A. & Merrill, E. H. First passage time analysis of animal movement and insights into the functional response. *Bull. Math. Biol.* (2009). doi:10.1007/s11538-008-9354-x

9. Hailey, A. & Coulson, I. M. Differential scaling of home-range area to daily movement distance

in two African tortoises. *Can. J. Zool.* (1996). doi:10.1139/z96-013

10. Loretto, D. & Vieira, M. V. The effects of reproductive and climatic seasons on movements in the black-eared opossum (Didelphis aurita Wied-neuwied, 1826). *J. Mammal.* (2005). doi:10.1644/beh-117.1

11. Pettorelli, N. *et al.* The Normalized Difference Vegetation Index (NDVI): Unforeseen successes in animal ecology. *Climate Research* (2011). doi:10.3354/cr00936

12. García-Ripollés, C., López-López, P., García-López, F., Aguilar, J. M. & Verdejo, J. Modelling nesting habitat preferences of Eurasian Griffon Vulture Gyps fulvus in eastern Iberian Peninsula. *Ardeola* (2005).

13. Donázar, J. A., Ceballos, O. & Cortés-Avizanda, A. Tourism in protected areas: Disentangling road and traffic effects on intra-guild scavenging processes. *Sci. Total Environ.* (2018). doi:10.1016/j.scitotenv.2018.02.186

14. Mateo-Tomás, P. & Olea, P. P. Livestock-driven land use change to model species distributions: Egyptian vulture as a case study. *Ecol. Indic.* (2015). doi:10.1016/j.ecolind.2015.05.017

15. Donovan, T. M. *et al.* Quantifying home range habitat requirements for bobcats (Lynx rufus) in Vermont, USA. *Biol. Conserv.* (2011). doi:10.1016/j.biocon.2011.06.026

16. Marzluff, J. M., Millspaugh, J. J., Hurvitz, P. & Handcock, M. S. Relating resources to a probabilistic measure of space use: Forest fragments and Steller's Jays. *Ecology* (2004). doi:10.1890/03-0114
